# Clinical features and therapeutic responses to proton pump inhibitor in patients with severe reflux esophagitis: A multicenter prospective observational study

**DOI:** 10.1002/jgh3.12455

**Published:** 2020-12-08

**Authors:** Kimio Isshi, Nobuyuki Matsuhashi, Takashi Joh, Kazuhide Higuchi, Katsuhiko Iwakiri, Takeshi Kamiya, Noriaki Manabe, Tatsuya Nakada, Maiko Ogawa, Seiji Arihiro, Ken Haruma, Koji Nakada

**Affiliations:** ^1^ Department of Gastroenterology Isshi Gastro‐Intestinal Clinic 2‐15‐21, Shinozaki‐cho Edogawa‐Ku 133‐0061 Tokyo Japan; ^2^ Department of Endoscopy The Jikei University School of Medicine 3‐15‐8, Nishishinbashi Minato‐Ku 105‐8461 Tokyo Japan; ^3^ Department of Gastroenterology NTT Medical Center Tokyo 5‐9‐22, Higashi‐Gotanda Shinagawa‐Ku 144‐8625 Tokyo Japan; ^4^ Department of Gastroenterology Gamagori City Hospital 1‐1, Mukaida Hirata‐cho Gamagori 443‐8501 Aichi Japan; ^5^ Second Department of Internal Medicine Osaka Medical College 2‐7, Daigakumachi Takatsuki 569‐8686 Osaka Japan; ^6^ Department of Gastroenterology Nippon Medical School Graduate School of Medicine 1‐1‐5, Sendagi Bunkyo‐Ku 133‐8603 Tokyo Japan; ^7^ Department of Medical Innovation Nagoya City University Graduate School Medical Sciences 1, Kwasumi Mizuhocho, Mizuho‐Ku Nagoya 467‐8601 Aichi Japan; ^8^ Division of Endoscopy and Ultrasonography, Department of Laboratory Medicine Kawasaki Medical School 2‐6‐1, Nakasange, Kita‐Ku Okayama 700‐8505 Okayama Japan; ^9^ Division of Gastroenterology and Hepatology, Department of Internal Medicine Katsushika Medical Center, The Jikei University School of Medicine 6‐41‐2, Aoto Katsushika‐Ku 125‐8506 Tokyo Japan; ^10^ Department of General Internal Medicine 2 Kawasaki Medical School Kawasaki Hospital 577, Matsushima Kurashiki 701‐0192 Okayama Japan; ^11^ Department of Laboratory Medicine The Jikei University School of Medicine 3‐25‐8, Nishishinbashi Minato‐Ku 105‐8461 Tokyo Japan

**Keywords:** complications, gastroesophageal reflux disease, modified Los Angeles classification, proton pump inhibitor, severe erosive reflux disease, therapeutic response

## Abstract

**Background and Aim:**

In patients with severe erosive reflux disease (ERD; Los Angeles classification grade C/D) who do not undergo endoscopic examination, insufficient strength and duration of proton pump inhibitor (PPI) therapy may lead to complications such as esophageal bleeding and stenosis. Therefore, to provide a safe and effective treatment for gastroesophageal reflux disease (GERD), we investigated the clinical features of patients with severe ERD and their responses to PPI therapy.

**Methods:**

Patients with GERD symptoms received PPI therapy for 4 weeks after endoscopic examination. The patients completed the Gastroesophageal reflux and dyspepsia therapeutic efficacy and satisfaction test questionnaire before and 2 or 4 weeks after PPI treatment. Patient characteristics, presence/absence of coexisting atrophic gastritis (AG) and hiatus hernia (HH), and responses to PPI therapy were compared in patients with GERD among three groups (nonerosive reflux disease, mild ERD [grade A/B], and severe ERD).

**Results:**

The severe ERD group had a significantly higher proportion of males, higher body mass index, and longer duration of GERD morbidity. Furthermore, the severe ERD group also had a significantly lower incidence of coexisting AG and higher incidence of HH. There was no difference in the severity of GERD before PPI treatment among the three groups. Unexpectedly, the response to PPI therapy was the best in the severe ERD group.

**Conclusion:**

Sufficient strength and period of PPI therapy are required, even if the symptoms show early improvement, when treating GERD patients without performing endoscopy, considering the possibility of severe ERD.

## Graphical abstract

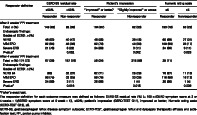

Outpatients with symptomatic gastroesophageal reflux disease (GERD) who will be prescribed proton pump inhibitor (PPI) treatment were recruited for this study. The patient characteristics, presence/absence of coexisting atrophic gastritis (AG) and hiatus hernia (HH), and responses to PPI therapy were compared in the patients with GERD, divided into three groups (nonerosive reflux disease, mild erosive reflux disease (ERD), and severe ERD). The severe ERD group also had a significantly lower incidence of coexisting AG and higher incidence of HH. There was no difference in the severity of the GERD symptoms before PPI treatment among the three groups. Unexpectedly, severe ERD patients had the greatest PPI treatment effect among the three groups.

## Introduction

Gastroesophageal reflux disease (GERD) is commonly encountered in clinical practice. The Japanese clinical practice guidelines for GERD allow proton pump inhibitor (PPI) therapy to be administered as the initial treatment, without performing endoscopy, in patients presenting with GERD symptoms.^1^ As a certain proportion of patients have severe erosive reflux disease (ERD), classified as grade C or D according to the endoscopic grading of GERD severity,^2^, ^3^ reducing PPI treatment on the basis of satisfactory relief in GERD symptoms at a comparatively early period is a matter of grave concern as it may lead to inadequate healing of mucosal injury, which could result in serious complications such as esophageal hemorrhage and stenosis.^4^, ^5^ Therefore, we examined the characteristics of the clinical features and therapeutic response of GERD symptoms to PPI therapy in patients with severe ERD.

## Methods

### *Study design*

This multicenter, prospective, observational study was conducted in 29 institutions in Japan; one or more investigators per institution were members of the GERD Society, a Japanese collaborative research group consisting of experts in the clinical practice of GERD. The study was conducted in accordance with the Declaration of Helsinki (sixth revision, 2008), after approval by the ethics committee of each institution or the central ethics committee of Nishi Clinic, Osaka, Japan. The study was registered with the University Hospital Medical Information Network Center Clinical Trials Registry in Japan (reference number [UMIN000006614]).

### *Patients*

Outpatients with symptomatic GERD who were prescribed PPI treatment in routine clinical care were recruited for this study. After upper gastrointestinal endoscopic examination, the patients were prescribed PPI therapy at the dosage level approved in Japan before the start of this study (April 2011). Inclusion criteria were as follows: (i) moderate or severe heartburn or acid regurgitation at least once a week or mild heartburn or acid regurgitation at least twice a week during the 2 weeks prior to the start of the study (Montreal Definition and Classification of GERD), (ii) patient age ≥20 years, and (iii) willingness to provide written informed consent. The exclusion criteria were (i) comorbidity or history of a disease that could potentially affect the results of the study (e.g. Zollinger‐Ellison syndrome, inflammatory bowel disease, irritable bowel syndrome, esophageal stricture, eosinophilic esophagitis, achalasia, malabsorption, or cerebrovascular disease); (ii) presence of concurrent symptoms of concern such as vomiting, peptic ulcer (except in the scarred stage), severe hepatic, renal and/or cardiac disease, mental disorder, uncontrolled metabolic disease, neurological disease, collagen disease, or other serious disease; (iii) confirmed or suspected malignancy; (iv) history of gastrointestinal tract resection or vagotomy; (v) history of hypersensitivity to PPIs or their excipients; (vi) history of *Helicobacter pylori* eradication therapy within 6 months prior to enrollment in the study; (vii) pregnancy, possible pregnancy, or breastfeeding; (viii) intake of a PPI or histamine type 2 (H2)‐receptor antagonist within 1 week prior to enrollment in the study; and (ix) patients otherwise deemed ineligible for enrolment in the study by the attending physician. Prohibited concomitant drugs were those that could affect the result of the study (PPIs other than the study drugs, H2 receptor antagonists, prokinetic agents, gastric mucosal protective agents, and anticholinergic drugs) and drugs that could interact with the study drugs.

### *Assessments*

Patients' demographic and clinical characteristics were recorded before the initiation of PPI therapy (0 week) using a series of questionnaires. The severity of reflux esophagitis was classified according to the modified Los Angeles (LA) classification system,^3^ and the incidences of coexisting atrophic gastritis (AG) and hiatus hernia (HH) were assessed by endoscopy. The responses of the GERD and dyspeptic symptoms to the treatment and the effect of the treatment on the quality of life (QOL) were assessed by comparing the results of the gastroesophageal reflux and dyspepsia therapeutic efficacy and satisfaction test (GERD‐TEST)^6^, ^7^ and an acute (1‐week recall) version of a health‐related QOL survey (SF‐8TM),^8^ respectively, recorded prior to the initiation of PPI therapy with those recorded after 2 and 4 weeks of PPI therapy. Psychiatric bias was assessed using the hospital anxiety and depression scale (HADS)^9^ before and at 4 weeks after the start of PPI therapy. The participants were instructed to respond to the questionnaires and send the completed questionnaires to our data center.

### *Questionnaires for data collection*

Patient characteristics were recorded using a questionnaire on patient's gender, age, height and weight, duration of GERD morbidity, and lifestyle factors (regularity of daily life, consumption of caffeine‐containing beverages or high‐fat meals, smoking status, and alcohol consumption). The GERD‐TEST is a questionnaire composed of 13 items used for investigating GERD and dyspepsia symptoms, their impact on daily life, and the impression of treatment efficacy. Questions Q 1–5 of the GERD‐TEST were intended to assess the severity of the upper abdominal symptoms; Q6–9 to assess the impact of the symptoms on the daily life activities of the patients, including eating, sleeping, other daily activities, and their mood; Q10–12 to evaluate the therapeutic responses to the PPI therapy; and Q13 to determine compliance with the medication. The responses to Q1–11 and Q13 were graded on a Likert scale and those to Q12 on a numeric rating scale (NRS) (Table 1).

**Table 1:** Gastroesophageal reflux and dyspepsia therapeutic efficacy and satisfaction test

Q1. Have you been bothered by heartburn during the past week? (By heartburn we mean a burning pain or discomfort behind the breastbone in your chest)
Q2. Have you been bothered by acid regurgitation during the past week? (By acid regurgitation we mean regurgitation or flow of sour or bitter fluid into your mouth)
Q3. Have you been bothered by epigastric pain or burning during the past week? (Epigastric pain includes any type of pain of the stomach)
Q4. Have you been bothered by postprandial fullness during the past week? (Postprandial fullness refers to discomfort or a sensation of heaviness caused by the food you consume remaining in the stomach)
Q5. Have you been bothered by early satiation during the past week? (Early satiation refers to the inability to finish a normally sized meal)
Response scale for Q1–5:
1 = no discomfort at all, 2 = slight discomfort, 3 = mild discomfort, 4 = moderate discomfort, 5 = moderately severe discomfort, 6 = severe discomfort, 7 = very severe discomfort.
Q6. During the past week, how often have you felt dissatisfaction because you were unable to eat meals as you intended due to chest and stomach symptoms? (Not being able to eat as you intended refers to the inability to eat the sufficient amount of food you want to eat at an uninhibited, natural pace)
Q7. During the past week, how often have you felt dissatisfaction due to impaired sleep caused by chest and stomach symptoms?
Q8. During the past week, how often have you felt dissatisfaction due to impairment of your work, housework, or other daily activities caused by chest and stomach symptoms?
Q9. During the past week, how often have you felt dissatisfaction because you were in a bad mood due to chest and stomach symptoms?
Response scale for Q6–9 1 = not at all, 2 = slightly, 3 = moderately, 4 = quite a lot, 5 = extremely
Q10. During the past week, how often have you wanted another drug in addition to the drug your doctor prescribed because of intense symptoms of heartburn and acid regurgitation?
1 = not at all, 2 = on 1 day, 3 = on 2 to 3 days, 4 **=** on 4 to 5 days, 5 = always.
Q11. During the past week, how have you felt about symptoms of heartburn and acid regurgitation as compared with the symptom severity before current treatment?
1 = extremely improved, 2 = improved, 3 = slightly improved, 4 **=** not changed, 5 **=** aggravated.
Q12. If 10 corresponds to your symptoms before current treatment and 0 is “symptom‐free”, what number corresponds to symptoms of heartburn and acid regurgitation during the past week?
Please circle the applicable score below:
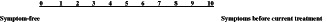
Q13. What proportion of the proton pump inhibitor prescribed to you did you take as instructed?
1 **=** took drug as instructed, 2 = generally took drug as instructed (took at least three‐quarters of the drug prescribed), 3 = sometimes forgot (took at least half but less than three‐quarters of the drug prescribed, 4 = took little (took less than half of the drug prescribed), 5 = did not take any.

Before therapy, questions about treatment efficacy and adherence (Q10–13) were excluded. The following scores were defined: GERD symptom score = (Q1 + Q2)/2, Epigastric pain/burning symptoms score = Q3, Postprandial distress symptom subscale = (Q4 + Q5)/2, Residual symptom rate (%) = 100 × (GERD symptom score at 4 weeks − 1)/(GERD symptom score at 0 week − 1).

The SF‐8 is a generic questionnaire used to investigate health status and comprises a physical component summary (PCS) and mental component summary (MCS). The HADS is a well‐established scale that measures psychiatric bias such as anxiety and depression, with each subscale composed of seven items.

### *Dose of* *PPI* *therapy*

After upper gastrointestinal endoscopy, the patients were treated with a PPI at the standard dose, that is, omeprazole or esomeprazole, 20 mg; lansoprazole, 30 mg; or rabeprazole, 10 mg or 20 mg once daily, for 4 weeks.

### *Definitions of the* *GERD* *symptom subscale*

The GERD symptom subscale (GERD‐SS) was defined as the mean of the scores for heartburn (Q1) and regurgitation (Q2).

### *Outcome measures*

To assess the therapeutic response to PPI therapy in patients with GERD, three outcome measures were used as follows: (i) the residual symptom rate of GERD‐SS, which was calculated as 100 × (GERD‐SS score at 2 or 4 weeks − 1)/(GERD‐SS score 0 week − 1). Thus, it was 100% when the GERD‐SS score at 2 or 4 weeks was equal to that at 0 weeks and 0% when the patient had no symptoms (a score of 1) at 2 or 4 weeks. The higher the residual symptom score, the poorer the response. (ii) Patient's impression of the treatment efficacy was another measure, which was scored on the basis of the response to Q11 of the GERD‐TEST (i.e. the score for the patient's impression of the improvement of the GERD symptoms compared with the severity noted before the start of the current treatment (1, markedly improved; 2, improved; 3, slightly improved; 4, unchanged; 5, aggravated)), and (iii) relative GERD symptom intensity based on the response to the NRS (Q12 of GERD‐TEST), quantified on an 11‐point scale (0 corresponding to freedom from symptoms [symptom‐free] to 10 corresponding to the severity of the symptoms before starting to take the current treatment). The definitions of responses for each outcome measure were as follows: (i) residual symptom rate ≤50%; (ii) patient's impression of improvement or better; and (iii) NRS ≤5.

### *Statistical analysis*

The data of the patients who underwent baseline endoscopic examination; answered the questionnaires within the defined period at 0, 2, and 4 weeks of treatment; provided information about gender, age, height, weight, and duration of GERD morbidity; responded to Q1–5 and Q11–13 of the GERD‐TEST; and showed a medication adherence rate of 75% were analyzed. The patients with GERD were divided into three groups according to the modified LA classification system: nonerosive reflux disease (NERD; grade N/M), mild ERD (grade A/B), and severe ERD (grade C/D). The statistical methods used to compare patient characteristics, endoscopic findings, and therapeutic efficacy among three groups included analysis of variance (anova) followed by Tukey's test and Fisher's exact test. Data analysis was conducted using JMP12.0.1 software (SAS Institute Inc., Cary, NC, USA). *P* values <0.05 were considered indicative of clinical significance.

## Results

### *Patient characteristics and* *GERD‐SS* *scores before the start of* *PPI* *therapy*

**Figure 1:**
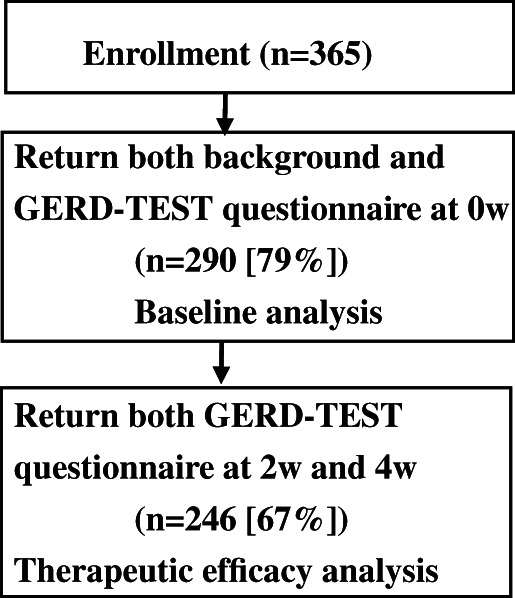
Flowchart of patient enrollment and data analysis. GERD‐TEST, gastroesophageal reflux and dyspepsia therapeutic efficacy and satisfaction test

A total of 365 patients were enrolled between April 2011 and July 2012. Data of 290 patients (79%) were analyzed at baseline and those of 264 patients (67%) at 2 or 4 weeks posttreatment (Fig. 1). At baseline, the mean age was 57.5 ± 13.9 years, with males comprising 61%. The mean body mass index (BMI) was 24.0 ± 3.9 kg/m^2^, the mean duration of GERD morbidity was 18.9 ± 30.1 months, and the mean GERD‐SS score was 3.4 ± 1.2. Endoscopic findings were classified as NERD (grade N/M: *n* = 107) in 36.9% of the patients and as ERD (grade A/B: *n* = 154 [53.1%], grade C/D: *n* = 29 [10%]) in 63.1% of patients (Table 2).

**Table 2:** Patient characteristics and GERD‐SS scores before the start of PPI therapy

	Endoscopic findings: Grades of GERD; *n* (%)					NERD *vs* Mild ERD	Mild ERD *vs* Severe ERD	NERD *vs* Severe ERD
	Total (*n* = 290)	NERD *n* = (107)	Mild ERD (*n* = 154)	Severe ERD (*n* = 29)	*P*‐value	*P*‐value	*P*‐value	*P*‐value
		36.9%	53.1%	10.0%				
Age (year)	57.5 ± 13.9	56.9 ± 15.0	57.1 ± 13.3	61.6 ± 12.9	0.239^a^			
Gender; *n* (%)					<0.001^b^			
Male	178 (61)	43 (40)	113 (73)	22 (76)				
Female	112 (39)	64 (60)	41 (27)	7 (27)				
BMI (kg/m^2^)	24.0 ± 3.9	22.7 ± 3.7	24.7 ± 3.7	25.4 ± 4.3	<0.001^a^	<0.001^c^	0.590^c^	0.002^c^
Duration of GERD								
Morbidity (months)	18.9 ± 30.1	14.3 ± 21.9	19.8 ± 30.4	34.2 ± 49.6	0.038^a^	0.494^c^	0.155^c^	0.031^c^
GERD‐SS	3.4 ± 1.2	3.6 ± 1.2	3.3 ± 1.3	3.3 ± 1.2	0.233^a^			

^a^ Analysis of variance.

^b^ Fisher's exact test.

^c^ Tukey test.

Los Angeles classification: Grades of GERD; *n* (%), NERD: Grade N; 62 (21%), Grade M; 45 (16%).

Mild ERD: Grade A 94 (32%); Grade B 60 (21%); Severe ERD: Grade C, 21 (7%); Grade D, 8 (3%), GERD‐SS = GERD‐TEST (Q1 + Q2)/2, Data are presented as means ± SD, .

ERD, erosive reflux disease; GERD, gastroesophageal reflux disease; GERD‐SS, GERD symptom subscale; GERD‐TEST, gastroesophageal reflux and dyspepsia therapeutic efficacy and satisfaction test; NERD, nonerosive reflux disease; PPI, proton pump inhibitor.

There were no significant differences in age among the three groups. The proportion of men was significantly higher in the mild ERD and severe ERD groups than in the NERD group (*P* = 0.001); the BMI was highest in the severe ERD group, followed by that in the mild ERD and NERD groups (*P* < 0.001); and the duration of GERD morbidity was significantly longer in the severe ERD group (*P* = 0.031). No difference was found in the GERD‐SS scores before the start of PPI therapy among the three groups (Table 2). Endoscopic examination of patients with GERD (*n* = 290) revealed AG in 49, 38, and 24% of the patients in the NERD, mild ERD, and severe ERD groups, respectively (*P* = 0.036), and HH in 17, 32, and 59% of the NERD, mild ERD, and severe ERD groups, respectively (*P* < 0.001), indicating that the incidence of AG was the lowest and that of HH was the highest in the severe ERD group (Table 3).

**Table 3:** Comparison of the incidence of coexisting atrophic gastritis and hiatal hernia among nonerosive reflux disease (NERD), mild erosive reflux disease (ERD), and severe ERD

Endoscopic findings: Grades of GERD; *n* (%)	Total (*n* = 290)	NERD (*n* = 107)	Mild ERD (*n* = 154)	Severe ERD (*n* = 29)	*P*‐value^a^
Accompanying endoscopic findings					
Atrophic gastritis (AG)					0.036
GERD with AG; *n* (%)	117 (40)	52 (49)	58 (38)	7 (24)	
GERD without AG; *n* (%)	173 (60)	55 (51)	96 (62)	22 (76)	
Hiatal hernia (HH)					<0.001
GERD with HH; *n* (%)	85 (29)	18 (17)	50 (32)	17 (59)	
GERD without HH; *n* (%)	205 (71)	89 (83)	104 (68)	12 (41)	

^a^ Fisher's exact test.

AG, atrophic gastritis; GERD, gastroesophageal reflux disease; HH, hiatal hernia; NERD, nonerosive reflux disease.

### *Comparison of therapeutic efficacy after 2 or 4 weeks of* *PPI* *therapy based on the* *GERD‐SS* *scores, patient's impression of the treatment efficacy, and the* *NRS* *among* *NERD, mild* *ERD,* *and severe* *ERD* *groups*

The response rates of patients with GERD to PPI therapy were as follows. Two weeks after the start of treatment, the response rates in the NERD, mild ERD, and severe ERD groups according to the residual symptom rate of GERD‐SS (*n* = 241) were 53, 64, and 78%, respectively (*P* = 0.062); those according to the patients' impression of the treatment efficacy (*n* = 246) were 55, 70, and 89%, respectively (*P* = 0.002); and those according to the NRS scores (*n* = 246) were 69, 83, and 89%, respectively (*P* = 0.023). Four weeks after the start of PPI therapy, the response rates in the NERD, mild ERD, and severe ERD groups according to the residual symptom rate of GERD‐SS were 63, 75, and 89%, respectively (*P* = 0.018); those according to the patients' impression of the treatment efficacy were 71, 75, and 96%, respectively (*P* = 0.015); and those according to the NRS scores were 87, 88, and 96%, respectively (*P* = 0.539) (Table 4). Despite the severe ERD group showing the most severe esophageal mucosal injury, it showed the highest response rates, according to all three parameters, at 2 and 4 weeks after the start of PPI therapy.

**Table 4:** Comparison of the therapeutic efficacy after 2 or 4 weeks of PPI therapy based on the GERD‐SS scores, patient's impression of the treatment efficacy, and the numeric rating scale among nonerosive reflux disease (NERD), mild erosive reflux disease (ERD), and severe ERD

Responder definition	GERD‐SS residual rate		Patient's impression		Numeric rating scale	
	≤50%	>50%	“Improved” or better?	“Slightly improved” or worse	≤5	>5
	Responder	Nonresponder	Responder	Nonresponder	Responder	Nonresponder
After 2 weeks' PPI treatment						
Total: *n* (%)	149 (62)	92 (38)	164 (67)	82 (33)	193 (78)	53 (22)
Endoscopic findings:						
Grades of GERD: *n* (%)						
NERD	46 (53)	40 (47)	48 (55)	39 (45)	60 (69)	27 (31)
Mild ERD	82 (64)	46 (36)	92 (70)	40 (30)	109 (83)	23 (17)
Severe ERD	21 (78)	6 (22)	24 (89)	3 (11)	24 (89)	3 (11)
*P*‐value^a^		0.062		0.002		0.023
After 4 weeks' PPI treatment						
Total: *n* (%)	174(72)	67(28)	187(76) (24)	59(24)	218(89)	28(11)
Endoscopic findings:						
Grades of GERD: *n* (%)						
NERD	54(63)	32 (37)	62 (71)	25 (29)	76 (87)	11 (13)
Mild ERD	96 (75)	32 (25)	99 (75)	33 (25)	116 (88)	16 (12)
Severe ERD	24 (89)	3 (11)	26 (96)	1 (4)	26 (96)	1 (4)
*P*‐value^a^		0.018		0.015		0.539

^a^ Fisher's exact test.

The responder definition for each outcome measure was defined as follows: GERD‐SS residual rate (%) [= 100 × (GERD symptom score at 2 or 4 weeks − 1)/(GERD symptom score at 0 week − 1)], ≤50%; patient's impression (GERD‐TEST Q11), improved or better; Numeric rating scale (GERD‐TEST Q12), ≤5.

GERD‐SS, gastroesophageal reflux disease symptom subscale; GERD‐TEST, gastroesophageal reflux and dyspepsia therapeutic efficacy and satisfaction test; PPI, proton pump inhibitor.

## Discussion

A certain proportion of patients presenting to hospital with a history of GERD symptoms has severe ERD.^5^, ^10^, ^11^ If these patients do not receive PPI treatment with sufficient strength and duration, the mucosal injury may not heal fully and may lead to complications such as esophageal bleeding and stenosis.^4^, ^5^, ^12^ Although studies have revealed the clinical features of severe ERD, further investigation is required for safer and more effective medical treatment of patients with GERD. In this study, the characteristics of severe ERD patients with respect to the severity of GERD symptoms at baseline and therapeutic response to PPI in addition to patients' background factors were examined. While there were no differences in the severity of the GERD symptoms at baseline among the NERD, mild ERD, and severe ERD groups, unexpectedly, the most marked symptomatic improvement following PPI treatment was observed in the severe ERD group. The results of the present study suggest that at least 8 weeks of usual PPI treatment is required, considering the probability of severe ERD, when PPI is administered as an initial treatment for symptomatic GERD patients without performing endoscopy.

The reported prevalence of ERD is approximately 10% in health checkup examinees in Japan.^1^ In addition, approximately half of the patients presenting to medical institutions with GERD symptoms have ERD.^13^ In turn, 14–24.2% of ERD patients are reported to have severe ERD (grade C/D).^5^, ^11^, ^14^ In the present study, of the patients presenting to the hospital with GERD symptoms and meeting the Montreal definition, 63% had ERD, and 10% (15.8% of the ERD patients) had severe ERD, consistent with previous reports. A considerable proportion of patients presenting to medical institutions with GERD symptoms have severe ERD.

Serious complications of GERD include hemorrhage, stenosis, and—rarely—perforation. The reported incidences of esophageal hemorrhage, esophageal stenosis, and esophageal hemorrhage with esophageal stenosis are 4.8, 2.6, and 0.8%, respectively, in the Japanese population.^5^ As these complications are associated with life‐threatening risks and can markedly reduce QOL, it is extremely important to prevent them while treating patients with GERD. Using multivariate analysis, Sakaguchi *et al*. explored the factors associated with the development of these complications and reported that advanced age and severe ERD were significant predictors of hemorrhage and that the use of psychotropic drugs and prolonged Barrett's esophagus, in addition to advanced age and severe ERD, were significant predictors of stenosis.

Obesity,^14^, ^15^, ^16^, ^17^, ^18^ male gender,^19^, ^20^, ^21^, ^22^, ^23^ advanced age,^23^, ^24^, ^25^, ^26^ smoking,^20^, ^21^, ^22^, ^26^, ^27^, ^28^, ^29^ lower prevalence of psychiatric disorders,^23^ and other factors have been reported to be associated with ERD including severe ERD. In addition, coexisting HH has also been reported as an endoscopic finding frequently associated with severe ERD.^16^, ^17^, ^18^, ^19^, ^20^, ^21^, ^22^, ^23^, ^24^, ^25^, ^26^, ^27^, ^28^, ^29^, ^30^

The results of our study also showed male gender, high BMI, long duration of GERD morbidity, low incidence of endoscopically detected coexisting AG, and presence of endoscopically detected coexisting HH as being associated with severe ERD; some of these factors have also been reported in previous studies. The severe ERD group had a significantly longer duration of GERD morbidity, suggesting that longer exposure to gastric acid may be involved in the progression to severe ERD.

GERD is considered an acid‐related disease, and PPI therapy has been shown to be remarkably effective.^31^ The severity of the mucosal injury was significantly correlated with the severity of the symptoms in patients with GERD,^2^ although many patients with severe ERD had mild or no symptoms.^2^, ^32^, ^33^ Some studies also reported that the severity of mucosal injury was not correlated with symptom severity.^32^, ^33^ The results of the present study also showed no significant differences in the severity of symptoms in patients with GERD among NERD, mild ERD, and severe ERD groups, suggesting that the severity of esophageal mucosal injury may not influence the severity of GERD symptoms before the start of PPI therapy. Unexpectedly, however, patients with severe ERD showed the highest response rates to PPI therapy. In other words, neither severe GERD symptoms nor a poor rate of improvement in GERD symptoms following PPI therapy was predictive of severe ERD. Therefore, in a patient with severe ERD, a reduction in the dose or duration of PPI therapy due to sufficient symptom improvement at the discretion of the physician or patient may result in serious complications such as esophageal hemorrhage and/or stenosis due to incomplete healing of the mucosal injury. This should be particularly borne in mind when the GERD patients are administered PPI therapy without performing endoscopy.

Although the reported healing rates of esophageal mucosal injury were 79–98% in all ERD patients who received PPIs in the present study at their usual doses for 8 weeks,^34^, ^35^, ^36^, ^37^, ^38^, ^39^, ^40^, ^41^, ^42^ the healing rate of esophageal mucosal injury after 8 weeks of PPI treatment has been reported to be as low as 45–77.5% in patients with severe ERD.^43^, ^44^, ^45^ It has also been reported that the healing rate of esophageal mucosal injury after 8 weeks of PPI therapy was significantly lower in patients with severe ERD than in those with mild ERD, indicating that the mucosal injury in cases of severe ERD takes longer to heal completely.^11^, ^46^ Therefore, long‐term continuous PPI treatment for more than 8 weeks, as well as later endoscopic examination, is recommended for patients with severe ERD.

In the treatment of GERD, it is recommended that upper gastrointestinal endoscopy be performed as soon as possible to confirm the presence of organic disease and severe ERD. When administering PPI therapy as initial treatment without endoscopy (particularly in male patients or those with a high BMI with a long duration of GERD morbidity, without previously endoscopically confirmed AG or HH), it is desirable to continue PPI therapy at above the usual doses for at least 8 weeks according to the Japanese clinical guidelines for GERD,^1^ considering the possibility of severe ERD, even if the GERD symptoms resolve early after the start of PPI therapy. In addition, if severe ERD is confirmed by endoscopy, further long‐term treatment with a drug that strongly suppresses acid secretion should be considered for the reliable cure of the mucosal injury and avoidance of complications such as hemorrhage and stenosis.

There are some limitations to this study. First, although the total number of patients was nearly 300, only 10% of the patients had severe ERD; therefore, the characterization of the clinical features of patients with severe ERD may not be entirely reliable. Second, as we did not perform endoscopy after the PPI therapy, the relationship between the degree of improvement of the symptoms and healing of the mucosal injury remains unclear in patients with severe ERD.
